# A framework for modelling soil structure dynamics induced by biological activity

**DOI:** 10.1111/gcb.15289

**Published:** 2020-08-23

**Authors:** Katharina Meurer, Jennie Barron, Claire Chenu, Elsa Coucheney, Matthew Fielding, Paul Hallett, Anke M. Herrmann, Thomas Keller, John Koestel, Mats Larsbo, Elisabet Lewan, Dani Or, David Parsons, Nargish Parvin, Astrid Taylor, Harry Vereecken, Nicholas Jarvis

**Affiliations:** ^1^ Soil and Environment Swedish University of Agricultural Sciences Uppsala Sweden; ^2^ UMR Ecosys INRA‐AgroParisTech Université Paris‐Saclay Thiverval‐Grignon France; ^3^ Stockholm Environment Institute SIANI‐SEI Stockholm Sweden; ^4^ School of Biological Sciences University of Aberdeen Aberdeen UK; ^5^ Agroecology and Environment Agroscope Zürich Switzerland; ^6^ Environmental Systems Science ETH Zürich Switzerland; ^7^ Agricultural Research for Northern Sweden Swedish University of Agricultural Sciences Umeå Sweden; ^8^ Ecology Swedish University of Agricultural Sciences Uppsala Sweden; ^9^ Bio‐ and Geo‐Sciences‐Agrosphere Forschungszentrum Jülich Jülich Germany

**Keywords:** biological processes, degradation, dynamics, modelling, soil, structure

## Abstract

Soil degradation is a worsening global phenomenon driven by socio‐economic pressures, poor land management practices and climate change. A deterioration of soil structure at timescales ranging from seconds to centuries is implicated in most forms of soil degradation including the depletion of nutrients and organic matter, erosion and compaction. New soil–crop models that could account for soil structure dynamics at decadal to centennial timescales would provide insights into the relative importance of the various underlying physical (e.g. tillage, traffic compaction, swell/shrink and freeze/thaw) and biological (e.g. plant root growth, soil microbial and faunal activity) mechanisms, their impacts on soil hydrological processes and plant growth, as well as the relevant timescales of soil degradation and recovery. However, the development of such a model remains a challenge due to the enormous complexity of the interactions in the soil–plant system. In this paper, we focus on the impacts of biological processes on soil structure dynamics, especially the growth of plant roots and the activity of soil fauna and microorganisms. We first define what we mean by soil structure and then review current understanding of how these biological agents impact soil structure. We then develop a new framework for modelling soil structure dynamics, which is designed to be compatible with soil–crop models that operate at the soil profile scale and for long temporal scales (i.e. decades, centuries). We illustrate the modelling concept with a case study on the role of root growth and earthworm bioturbation in restoring the structure of a severely compacted soil.

## Graphical abstract

This photograph, depicting ant bioturbation, was taken at the compaction recovery experiment at Agroscope, Zurich, Switzerland. Together with other biological processes, faunal bioturbation profoundly influences soil structure and thus soil physical and hydraulic properties, hydrological processes and plant growth. The parsimonious model concept developed in this paper, which is designed to be compatible with profile‐scale soil–crop models, allows simulation of the effects of biological agents (e.g. plant roots and soil‐living organisms) on soil structure dynamics.

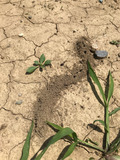

## 1 INTRODUCTION

**FIGURE 1:**
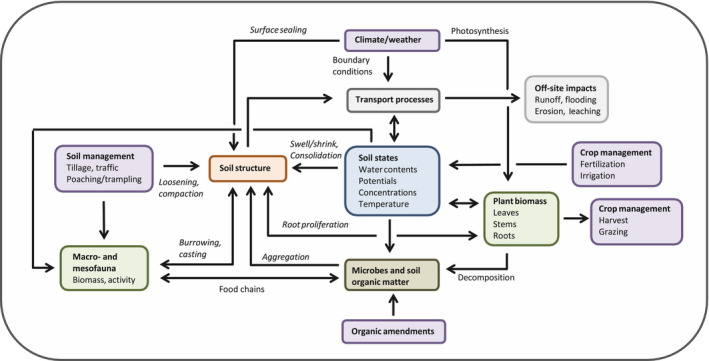
Schematic diagram of the drivers, agents and processes (italicized) governing the dynamics of soil structure and its effects. Arrows indicate directions of influence

**FIGURE 2:**
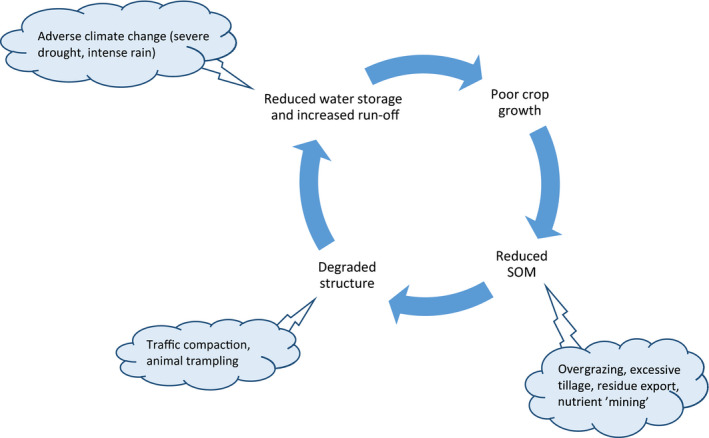
Positive feedback loops driving soil structure degradation in cropping systems driven by adverse climatic changes and non‐sustainable land use and management practices

The physical arrangement of the soil pore space (‘soil structure’) profoundly influences life in soil (e.g. root growth and microbial activity) and many important processes (e.g. rates of water and air movement, solute leaching, carbon and nutrient cycling, water and nutrient uptake by crops) and thus the ecosystem services that soil can deliver (e.g. Bünemann et al., 2018; Dominati, Patterson, & Mackay, 2010; Keesstra et al., 2016; Powlson et al., 2011; Robinson, Lebron, & Vereecken, 2009). The structure of soil is constantly evolving, driven by changes in exogeneous factors (i.e. climate and land management) mediated by various biological (e.g. root growth, microbial and faunal activity) and physical processes (e.g. swell‐shrink, freeze–thaw; Figure 1) that span timescales ranging from seconds (e.g. traffic compaction) to decades and centuries (e.g. depletion or accumulation of soil organic matter, SOM). In the long term, adverse changes in climate or the adoption of non‐sustainable land management practices may degrade the structure of soil to such an extent that it becomes unsuitable for crop production (e.g. Food & Agriculture Organization of the United Nations [FAO/ITPS], 2015; Gregory et al., 2015; Intergovernmental Panel on Climate Change [IPCC], 2019; Intergovernmental Science‐Policy Platform on Biodiversity and Ecosystem Services [IPBES], 2018; Rickson et al., 2015; Smith et al., 2016). Figure 2 illustrates how positive feedback mechanisms between plant growth, soil structure and hydrological processes can lead to a vicious circle of soil degradation driven by either land use or climate change (e.g. D’Odorico, Bhattachan, Davis, Ravi, & Runyon, 2013; Young et al., 1998). Recent global assessments suggest that the majority of the soils of the world are now in very poor, poor, or only fair physical condition (FAO/ITPS, 2015) and that without effective mitigation, this situation will worsen due to a combination of climate change and increased pressures on the land (IPBES, 2018; IPCC, 2019).

Soil–crop models operating at the plot and field scales are widely used to evaluate the effects of land use and management practices (e.g. crop rotations, tillage, irrigation, fertilization) and climate change on crop production and environmental quality (e.g. Bergez et al., 2014; Brilli et al., 2017; Constantin et al., 2019; Dilla, Smethurst, Barry, Parsons, & Denboba, 2018; Eckersten et al., 2012; Robertson, Rebetzke, & Norton, 2015). Existing models can be used to quantify the effects of soil degradation by running scenario simulations with contrasting soil physical and hydraulic properties. For example, Cresswell, Smiles, and Williams (1992) simulated the effects of alternative tillage systems and the presence of surface crusts and plough pans on surface runoff generation with the hydrological model SWIM. However, such an approach cannot provide any insights into the underlying mechanisms and timescales of soil degradation and recovery. The individual processes driving soil structure dynamics are well known (Dexter, 1991; Kay, 1990; Oades, 1993; Young et al., 1998). Nevertheless, there are few instances of treatments of soil structure dynamics being incorporated into soil–crop models, even though its importance has long been recognized (Connolly, 1998). Accounting for soil structure dynamics at decadal to centennial scales in soil–crop models would enable quantification of the potential for management practices to alleviate soil degradation, as well as estimation of the timescales of recovery (Kibblewhite, Chambers, & Goulding, 2016). However, the development of such a model remains a challenge due to the enormous complexity of process interactions in the soil–plant system (Hallett, Karim, Bengough, & Otten, 2013; Vereecken et al., 2016; Vogel et al., 2018). Empirical descriptions of seasonal variations of soil physical and hydraulic properties induced by tillage and subsequent consolidation have been employed in some model applications (e.g. Alletto et al., 2015; Chandrasekhar et al., 2018; Maharjan et al., 2018; Or, Leij, Snyder, & Ghezzehei, 2000; Schwen, Bodner, & Loiskandl, 2011; Strudley, Green, & Ascough II, 2008). Tillage (e.g. ploughing) lifts, loosens and fragments the soil, which increases the soil volume and creates larger soil voids, thereby improving some soil functions in topsoil. However, the benefits of tillage are usually rapidly lost since the loose structure is unstable and collapses (Hao et al., 2011). Therefore, tillage alone cannot completely restore the physical structure of a soil damaged, for example, by traffic compaction. This is especially the case at plough depth and in the subsoil (e.g. Alakukku, 1996; Arvidsson & Håkansson, 1996; Dexter, 1991; Weisskopf, Reiser, Rek, & Oberholzer, 2010). Biological agents and processes are particularly important for the maintenance of soil structure and the recovery of degraded soils (Angers & Caron, 1998; Colombi & Keller, 2019; Dexter, 1991; Kay, 1990; Six, Bossuyt, Degryze, & Denef, 2004; Young et al., 1998), yet no work has been done so far towards incorporating soil structure dynamics induced by biological processes into soil–crop models.

Therefore, in this paper, we focus on the impacts of biological processes and agents on the dynamics of soil structure, in particular, the growth of plant roots and the activity of soil‐living organisms. In the following, we first define soil structure and then review current understanding of how biological agents and processes govern soil structure dynamics, with particular emphasis on insights gained from applying modern imaging techniques under controlled experimental conditions. We then present a new concept for modelling soil structure dynamics that should be compatible with soil–crop models commonly used to evaluate the effects of management practices on crop production and the environment. Finally, the potential for applications of the concept is illustrated by a case study on the role of root growth and earthworm bioturbation in restoring the structure of a severely compacted soil.

## 2 SOIL STRUCTURE AND SOIL STRUCTURE DYNAMICS: OVERVIEW AND SOME FUNDAMENTAL CONCEPTS

From a linguistic point of view, structure refers to the arrangement of elements in a body or object. In the context of soils, structure can be defined as the spatial arrangement of mineral particles, organic material and pore spaces in soil (e.g. Dexter, 1988; Oades, 1993). Soil structure dynamics result either from changes in the mass of solids in soil or from energy inputs and resulting mechanical forces that cause particle displacement. The ability of soil to resist these applied stresses is termed strength or critical stress. This resistance to deformation depends partly on the structure itself. The energy input that modifies structure by displacing particles derives from both abiotic and biotic sources (Lin, 2011; see Figure 1). Abiotic sources of energy include the action of tillage implements and vehicle wheels, the kinetic energy in rainfall and the potential energy associated with air–water interfaces in soil. The biotic sources result from the growth of plant root systems and the activity of soil fauna, all powered by the conversion of solar energy into organic matter (Lavelle et al., 2016; Young et al., 1998).

Some changes in soil structure may be essentially irreversible at human timescales, with the soil evolving towards ‘alternative stable states’ (Robinson et al., 2016, 2019). The change in soil structure occurring after drainage of waterlogged clay soils is one well‐known example (e.g. Ellis & Atherton, 2003; Kim, Vereecken, Feyen, Boels, & Bronswijk, 1992). In other cases, structural changes may be reversible although the timescales of degradation and recovery can be very different. For example, subsoil compaction due to heavy vehicle traffic occurs during seconds, whilst recovery to pre‐compaction conditions by natural processes is usually very slow, taking decades or even centuries (e.g. Alakukku, 1996; Etana et al., 2013; Nawaz, Bourrié, & Trolard, 2013; Schlüter et al., 2018; Webb, 2002). In contrast, the recovery of some soil functions in degraded topsoil resulting from biological agents and processes (e.g. root growth and macro‐faunal activity) can sometimes be surprisingly fast (i.e. from weeks and months to just a few years; e.g. Blanchart, Albrecht, Chevallier, & Hartmann, 2004; Brown, Scholtz, Janeau, Grellier, & Podwojewski, 2010; Capowiez et al., 2012; Drewry, 2006; Fell, Matter, Keller, & Boivin, 2018; Fischer et al., 2015; Lucas, Schlüter, Vogel, & Vetterlein, 2019; McLenaghen, Malcolm, Cameron, Di, & McLaren, 2017; Obi, 1999).

**FIGURE 3:**
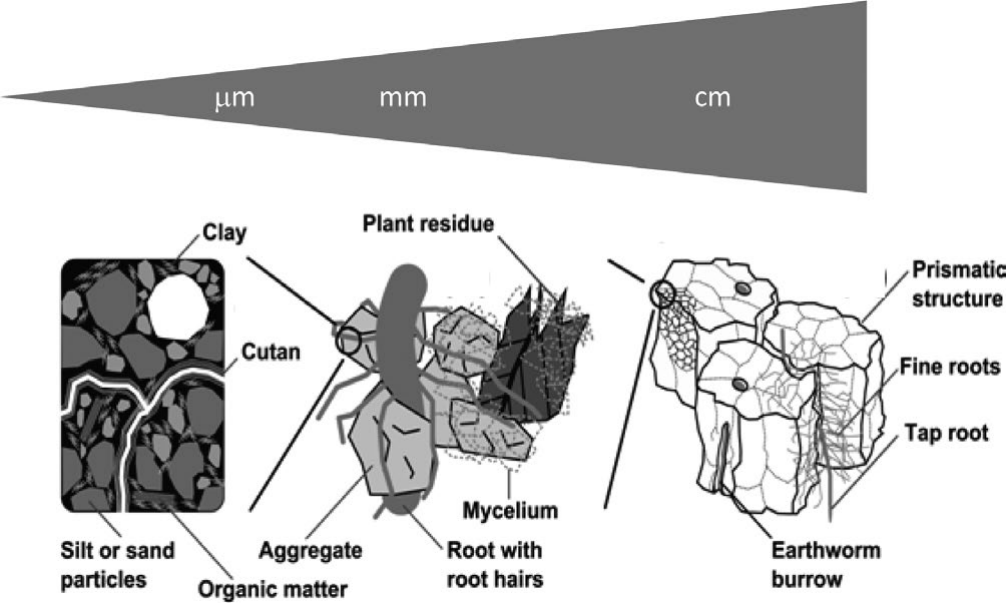
Soil structural organization across scales

The diversity of structure‐forming processes and agents means that natural soil is structured across a very wide range of scales (Hallett et al., 2013; Vogel & Roth, 2003; Young et al., 1998; Figure 3). Physical forces (e.g. soil tillage, swell/shrink, freeze–thaw) produce cracks and soil fragments at the millimetre or even centimetre scale that are easily visible to the naked eye (e.g. Emmet‐Booth, Forristal, Fenton, Ball, & Holden, 2016; Franco, Guimarães, Tormena, Cherubin, & Favilla, 2019; Mohammed, Hirmas, Nemes, & Giménez, 2020). Biological agents and processes maintain soil structure across a very broad range of scales (Figure 3). These range from large millimetre‐sized biopores created by plant roots and soil macro‐fauna to aggregation at the micrometre scale resulting from the activity of microorganisms decomposing fresh OM supplied to soil. This scale dependence of soil structure is often expressed in terms of a hierarchy consisting of two or more characteristic scales (e.g. micro‐ and macro‐aggregates, or soil matrix and macropores; Durner, 1994; Jarvis & Larsbo, 2012; Six et al., 2004; Vogel & Roth, 2003). Without a continual input of fresh organic material, the SOM store would be depleted, life in soil would cease and the multiscale (hierarchical) structure of soil would tend to degrade towards a state of increasing disorder or entropy (e.g. Feeney et al., 2006; Lavelle et al., 2006, 2016; Lin, 2011; Oades, 1993). In this case, the matrix porosity would decline towards a minimum value determined by the closest possible particle packing, while the pore size distribution would closely mirror the particle size distribution (Arya, Leij, van Genuchten, & Shouse, 1999). A porous medium consisting of randomly packed particles also has a structure defined by the geometry and topology of its pore space and solids. This suggests that in the context of physical soil quality, we should distinguish between an inherent ‘textural’” pore space and the ‘structural’ pore space generated by various abiotic processes and biological agents (Figure 1; Childs, 1969; Dexter, 2004; Fies & Stengel, 1981; Reynolds, Drury, Tan, Fox, & Yang, 2009; Yoon & Gimenéz, 2012).

Soil structure can be quantified with a wide range of metrics, either from the perspective of the spatial arrangement of the soil solid or its complement, the soil pore space. In our review and modelling we focus on characteristics of the soil pore space as it enables natural links between structure and flow and transport processes in soil (Rabot, Wiesmeier, Schlüter, & Vogel, 2018; Young, Crawford, & Rappoldt, 2001). The Minkowski functions represent a concise way to describe the geometry and topology of a multiscale binary medium (Vogel, Weller, & Schlüter, 2010), being defined as the volume and connectivity of each phase and the surface area and curvature of their interface, expressed as a function of pore diameter. These functions reflect complementary aspects of soil structure and should therefore have relevance for many different processes in soils (San José Martínez, Martín, & García‐Gutiérrez, 2018; Vogel et al., 2010). For example, the surface area controls the interactions of solutes between water‐filled pores and solid surfaces, while the curvature gives information on pore shape, which may be relevant for soil mechanical properties (Vogel et al., 2010). The pore volume fraction as a function of pore diameter (the pore size distribution of soil) is an especially important relationship as it exerts a strong control on soil‐living organisms (Young et al., 1998). It also regulates the storage and flow of water in soil, forming the basis for the water retention and hydraulic conductivity functions employed in hydrological models based on Richards’ equation. Flow and transport processes in soil are also influenced by pore space connectivity, especially for larger macropores (Jarvis, Koestel, & Larsbo, 2016). This is because the connectivity of pore space of a given minimum diameter is strongly determined by its fractional volume and macropores are relatively sparsely distributed (Jarvis, Larsbo, & Koestel, 2017; Koestel, Larsbo, & Jarvis, 2020; Schlüter et al., 2018).

## 3 BIOLOGICAL AGENTS OF SOIL STRUCTURE DYNAMICS

The structure of soil is profoundly altered by plant root growth, the movement and feeding activity of soil fauna and by soil microorganisms decomposing the organic matter supplied to the soil as plant litter, root exudates and organic amendments. These processes are described in the following sections.

### 3.1 Root growth

Plants directly alter the soil pore space through the growth of roots through the soil matrix, which induces particle displacement, predominantly axially in front of the root and radially beside the root (e.g. Keyes et al., 2016; Koestel & Schlüter, 2019; Vollsnes, Futsaether, & Bengough, 2010), thereby compressing pre‐existing pores (e.g. Aravena, Berli, Ghezzehei, & Tyler, 2011; Ruiz, Or, & Schymanski, 2015; Ruiz, Schymanski, & Or, 2017). The subsequent decay of roots creates vertically extensive, well‐connected structural pore networks (e.g. Hellner, Koestel, Ulén, & Larsbo, 2018; Lucas et al., 2019; Luo, Lin, & Halleck, 2008; Luo, Lin, & Li, 2010; Pagenkemper et al., 2015; Pagenkemper, Peth, Puschmann, & Horn, 2013). In a comparison of 12 cover crop species, Bodner, Leitner, and Kaul (2014) found that plants with coarser roots increased soil macroporosity, whereas those with dense fine root systems induced a more heterogeneous pore size distribution with larger microporosity (pores <15 μm in diameter). By inferring soil hydraulic properties from drainage experiments on laboratory soil columns, Scholl et al. (2014) demonstrated how the growth of plant roots altered soil pore size distributions, with increases in the volumes of pores <2.5 μm and >500 μm in diameter. In a field experiment, Pires et al. (2017) showed that elimination of weeds from the inter‐rows of a coffee crop, by either mechanical methods or herbicide application, reduced the volume of pores with diameters larger than 25 μm. Other field experiments have shown that plant species with large tap roots have the potential to restore some of the physical functions of degraded subsoil by creating large biopores (Cresswell & Kirkegaard, 1995; Meek, DeTar, Rolph, Recher, & Carter, 1990; Uteau, Pagenkemper, Peth, & Horn, 2013; Yunusa & Newton, 2003).

Plant roots also exert important indirect controls on soil structure formation through plant water uptake and soil shrinkage (Jotisankasa & Sirirattanachat, 2017; Kay, 1990) and by increasing the stability of the soil to resist mechanical stresses (e.g. Bearden, 2001; Chen et al., 2019; Hallett et al., 2009; Kohler‐Milleret, Le Bayon, Chenu, Gobat, & Boivin, 2013; Milleret, Le Bayon, Lamy, Gobat, & Boivin, 2009). They are also a major source of the organic carbon supplied to soil (Haichar, Heulin, Guyonnet, & Achouak, 2016; Jones, Nguyen, & Finlay, 2009), which drives the activity and growth of soil microorganisms and fauna, and thus the development of an aggregated soil structure. In addition to enhancing nutrient and water capture by plants and influencing microbial populations at the root–soil interface, the organic exudates and mucilages secreted by roots are known to affect soil structure (Benard et al., 2019; York, Carminati, Ritz, & Bennett, 2016). Although polysaccharides produced by roots improve aggregation by gelling soil particles, organic acids can have a dispersive effect that liberates trapped nutrients and eases root penetration (Naveed et al., 2017; Oleghe, Naveed, Baggs, & Hallett, 2017). In terms of soil structure dynamics, the impacts of root exudates are rapid and underpin the formation of the thin zone at the root–soil interface termed the rhizosphere (York et al., 2016). Over relatively short time periods, root exudates and mucilages are transformed by microorganisms into organic compounds (Jones et al., 2009) that generally stabilize soil structure (Baumert et al., 2018; Naveed et al., 2017).

### 3.2 Soil fauna

Soil macrofauna such as termites, ants, beetles and earthworms dramatically alter the physical architecture of their habitat, the pore space of the soil (Blanchart, Lavelle, Braudeau, Le Bissonais, & Valentin, 1997; Jones, Lawton, & Shachak, 1994; Lavelle, 2002; Lavelle et al., 2006, 2016; Nichols et al., 2008), both by displacing soil particles by their movement through soil and by ingesting soil to extract organic material as a food source (e.g. Curry & Schmidt, 2007; McKenzie & Dexter, 1988; Ruiz et al., 2015, 2017; Taylor, Lenoir, Vegerfors, & Persson, 2018). Although their activities and impact have been much less extensively studied (e.g. Maaß, Caruso, & Rillig, 2015), soil mesofauna (i.e. soil animals with a diameter between 0.1 and 2 mm) are also known to affect soil structure (Wolters, 2001) albeit at a smaller scale commensurate with their body size. In most soils, the most abundant mesofaunal groups are the mites, springtails (collembolans) and potworms (enchytraeids).

In a similar way as for plant root growth, the burrowing activity of soil macrofauna displaces soil particles and compresses the surrounding pore space (e.g. Binet & Curmi, 1992; Capowiez, Sammartino, & Michel, 2011; Koestel & Schlüter, 2019; Rogasik, Schrader, Onasch, Kiesel, & Gerke, 2014; Schrader, Rogasik, Onasch, & Jégou, 2007; West, Hendrix, & Bruce, 1991). Barnett, Bengough, and McKenzie (2009) studied the dynamics of soil displacement by two deep‐burrowing anecic earthworm species, both of which caused mostly radial soil displacement and very little axial movement, with *Lumbricus terrestris* displacing significantly more soil than *Aporrectodea longa*. Ruiz et al. (2017) compared soil drilling by plant roots and earthworms and concluded that earthworms must withstand twice the stress to penetrate soil relative to plant roots, because of their much faster movement. They found that as soil dries, increasing soil strength would impede earthworm activity long before inhibiting plant root growth. The critical water content that would begin to inhibit earthworm movement by particle displacement was estimated to be close to field capacity (i.e. the water content at a pressure potential of −33 kPa).

Under favourable environmental conditions, up to c. 20%–25% of the total topsoil mass can be ingested each year by earthworms, predominantly by endogeic species (e.g. Anderson, 1988; Curry & Schmidt, 2007). The soil ingested by earthworms resides in the gut for some time and can therefore be egested at some distance from the site of ingestion, not only within the soil profile, but also at the surface. Such directed (non‐random) and non‐local transport of soil particles due to earthworm bioturbation can alter bulk densities in the soil profile (e.g. Jarvis, Taylor, Larsbo, Etana, & Rosén, 2010). Thus, several microcosm experiments have demonstrated the loosening of compacted soil by earthworm activity (e.g. Francis, Tabley, Butler, & Fraser, 2001; Joschko, Diestel, & Larink, 1989; Ponder, Li, Jordan, & Berry, 2000; Zund, Pillai‐McGarry, McGarry, & Bray, 1997). Dramatic changes in soil porosity and/or bulk density have also been demonstrated following accidental earthworm invasions or by their deliberate introduction (inoculation) or elimination using toxic substances (e.g. Alegre, Pashanasi, & Lavelle, 1996; Baker, Brown, Butt, Curry, & Scullion, 2006; Barros, Curmi, Hallaire, Chauvel, & Lavelle, 2001; Chauvel et al., 1999; Clements, Murray, & Sturdy, 1991; Hallam et al., 2020). Other field studies have reported significant spatial correlations between bulk density and the composition of earthworm communities (Decaëns & Rossi, 2001; Rossi, 2003).

Earthworm species produce casts with a characteristic porosity that may differ from the ingested soil (e.g. Blanchart, 1992; Blanchart, Bruand, & Lavelle, 1993; Blanchart et al., 1997; Chauvel et al., 1999; Decaëns, 2000). The pore size distribution of casts may also differ significantly from that of the original ingested soil (e.g. Blanchart et al., 1993; Görres, Savin, & Amador, 2001; Jouquet, Bottinelli, Podwojewski, Hallaire, & Tran Duc, 2008; Lipiec, Turski, Hajnos, & Świeboda, 2015). This implies that in addition to creating macropores by burrowing (Capowiez, Pierret, Daniel, Monestiez, & Kretzschmar, 1998; Joschko et al., 1991; Pagenkemper et al., 2015), casting by earthworms can alter the pore size distribution and water retention properties of the soil matrix. Passage through the gut of earthworms also alters several other important biochemical, physical and mechanical properties of the ingested soil, including the organic carbon content, tensile strength, stability in water and water repellency (Barré, McKenzie, & Hallett, 2009; Jouquet et al., 2008; Larink, Werner, Langmaack, & Schrader, 2001; Lipiec et al., 2015; Schrader & Zhang, 1997; van Groenigen et al., 2019). Changes in the physical and mechanical properties of soil induced by ingestion and casting can have important consequences for soil aggregation and structural stability and therefore the temporal evolution of bulk density and porosity (e.g. Barré et al., 2009; Jouquet, Huchet, Bottinelli, Thu, & Duc, 2012; Larink et al., 2001).

Just like the larger earthworms, the burrowing activity of smaller enchytraeid worms creates soil pores of a similar diameter to their body width (c. 0.50 and 0.75 mm; Didden, 1990; Porre, van Groenigen, De Deyn, de Goede, & Lubbers, 2016). Enchytraeids ingest much less soil (<0.01% of the bulk soil mass per year; Didden, 1990) than the larger earthworms and only in the uppermost soil layers. However, their activity has been shown to significantly affect soil pore size distribution, pore continuity and soil aeration (e.g. Didden, 1990; Porre et al., 2016). Much less is known about the effects of mites and collembola on soil structure (Maaß et al., 2015). The presence of collembola has been shown to increase water‐stable aggregation in laboratory experiments (Siddiky, Kohler, Cosme, & Rillig, 2012; Siddiky, Schaller, Caruso, & Rillig, 2012). However, Porre et al. (2016) found no significant effects of mites on soil pore structure quantified by X‐ray tomography. Earlier micromorphological studies showed that mesofaunal faecal pellets typically c. 50–200 µm in diameter can be a major component of aggregated soils (e.g. Boersma & Kooistra, 1994; Dawod & FitzPatrick, 1993; Topoliantz, Ponge, & Viaux, 2000). Soil macro‐ and mesofauna also indirectly regulate soil structure dynamics through their impacts on the growth and activity of the microbial populations that maintain microscale aggregation in soil (e.g. Angst et al., 2017; Görres et al., 2001; Lubbers, Pulleman, & van Groenigen, 2017; Maaß et al., 2015; Medina‐Sauza et al., 2019).

### 3.3 Soil microorganisms and SOM

Natural soils are characterized by an aggregated structure that is, in part, generated and stabilized by the growth and activity of soil‐living bacteria and fungi (e.g. Chenu & Cosentino, 2011; Young & Crawford, 2004). Controlled manipulation experiments using initially sieved and repacked soils have demonstrated that microbial turnover of added organic carbon can increase porosity and pore network connectivity and alter the pore size distribution at timescales of only a few weeks (e.g. Bucka, Kölbl, Uteau, Peth, & Kögel‐Knabner, 2019; de Gryze et al., 2006; Feeney et al., 2006). In most of these experiments, soil water contents were maintained by water addition every 1 or 2 days, so that some of the observed changes in soil structure may be attributable to swelling and shrinkage during wetting and drying cycles (de Gryze et al., 2006). In this respect, the physical and biological processes of structure formation act synergistically. Thus, microorganisms modify the properties of their immediate environment by exuding extracellular polymeric substances and it has been shown that microcracks appear at the boundaries of these micro‐environments on wetting and drying (e.g. Chenu & Cosentino, 2011; Robert & Chenu, 1992). In their experiments, Bucka et al. (2019) eliminated the effects of wetting and drying cycles by incubating samples at a constant pressure head of −150 cm. Thus, other forces must have caused the rearrangement of soil particles and changes in soil structure found in their study. These may include positive gas pressures resulting from the microbial production of CO_2_ (Bucka et al., 2019; Helliwell, Miller, Whalley, Mooney, & Sturrock, 2014) and the growth and movement of fungal hyphae (Bearden, 2001; de Gryze et al., 2006; Dorioz, Robert, & Chenu, 1993). The aggregated structure of soil is stabilized by microbial exudation of hydrophobic extracellular proteins and polysaccharides (e.g. Baumert et al., 2018; Chenu & Cosentino, 2011; Hallett & Young, 1999) and enmeshment by fungal hyphae (Chenu & Cosentino, 2011).

A proportion of the fresh organic matter turned over by microorganisms is retained in the soil rather than being mineralized. Microbially processed organic matter stabilizes the aggregated structure by complexation with clay minerals and iron and aluminium oxides (e.g. Chenu & Cosentino, 2011; Dignac et al., 2017; Tisdall & Oades, 1982). SOM also significantly affects the mechanical properties of soil and thus the stability of the structure of the soil to applied mechanical stresses. SOM is known to influence soil swell–shrink behaviour (Boivin, Schäffer, & Sturny, 2009) and soil strength, friability and workability (Arthur, Schjønning, Tuller, & de Jonge, 2013; Chenu & Guérif, 1991; Gregory et al., 2009; Obour, Jensen, Lamandé, Watts, & Munkholm, 2018; Watts & Dexter, 1998). SOM also tends to decrease soil wettability (e.g. Chenu, Le Bissonais, & Arrouays, 2000). Thus, SOM increases aggregate stability during wetting, as a consequence of both increases in interparticle bonding strength and decreased wettability (Chenu et al., 2000; Hallett et al., 2013; Sarker et al., 2018).

It is by now well‐established that, as a consequence of the various interacting physical and biological mechanisms discussed above, soils of larger organic matter content generally have larger porosities (e.g. Federer, Turcotte, & Smith, 1993; Haynes & Naidu, 1998; Jarvis, Forkman, et al., 2017; Johannes et al., 2017). Indeed, some studies found that an increase in the mass or volume of SOM tends to increase the soil pore volume in an approximately linear fashion (e.g. Boivin et al., 2009; Emerson & McGarry, 2003; Johannes et al., 2017). In contrast, the effects of SOM on the pore size distribution and thus soil water retention, are still a subject of some controversy. Hudson (1994) found that within broad textural classes, SOM content significantly increased the plant available water capacity, with the water stored at field capacity (the water content at a pressure potential of −33 kPa) increasing more than at the wilting point. Conversely, other studies found only limited effects on the soil pore size distribution and water retention curve (e.g. Libohova et al., 2018; Loveland & Webb, 2003; Minasny & McBratney, 2018; Pituello, Dal Ferro, Simonetti, Berti, & Morari, 2016; Rawls, Pachepsky, Ritchie, Sobecki, & Bloodworth, 2003). These studies show that SOM is associated with increases in soil water storage at all pressure heads in the plant available water range, although usually somewhat more so at pressure heads close to field capacity.

## 4 CONCEPTS FOR MODELLING SOIL STRUCTURE DYNAMICS

Alongside the experimental research discussed in the foregoing, some detailed process‐oriented models have also been developed that describe interactions between soil structure and various individual biological agents such as roots, earthworms or microorganisms (e.g. Baumert et al., 2018; Blanchart et al., 2009; Chakrawal et al., 2020; Ebrahimi & Or, 2016; Hallett et al., 2013; Monga et al., 2014; Roose et al., 2016; Ruiz et al., 2017). Although such approaches lead to valuable insights into the individual governing processes, they operate at small spatial (e.g. soil aggregates or the soil surrounding a single root or earthworm) and temporal scales (days, weeks, seasons). In addition to continuing and intensifying this fundamental research to improve process understanding at the microscale (Hallett et al., 2013; Vereecken et al., 2016), it should also be profitable to focus efforts on developing simpler empirical model concepts for soil structure dynamics informed by this process‐oriented research. This kind of heuristic model would be compatible with the soil–crop models that are applicable at the spatial and temporal scales relevant for soil and crop management (e.g. soil profiles, decades and centuries). Ideally, such an empirical approach to modelling soil structure dynamics would integrate the current understanding of a range of different governing processes within a single conceptual model framework. This would enable model users to assess the relative importance of individual processes and their characteristic timescales, as well as impacts on crop performance and environmental quality. In this context, the challenge is to capture the considerable complexity of the various governing processes with a relatively simple concept in order to minimize the number of additional parameters required, whilst retaining a sufficient degree of realism. Model parsimony is critical because the available experimental data will likely be insufficient to unequivocally parameterize complex models (Beven, 2006; Bradford, 2016; Luo, Wang, & Sun, 2017).

Modelling temporal variations in pore size distribution and porosity is a simple, yet a potentially powerful way to account for soil structure dynamics (Or et al., 2000), because these properties regulate the habitat for soil‐living organisms and also determine the hydraulic functions that are fundamental to soil water flow and storage as well as plant water uptake and growth. For this reason, the shape of the soil water retention curve has been considered as a useful indicator of soil physical quality (Dexter, 2004; Reynolds et al., 2009). One simple way to generate dynamic soil water retention curves would be to use pedotransfer functions (e.g. Keyvanshokouhi et al., 2019). However, such a statistical approach would have quite limited applicability, since the only time‐variable properties generally available in the soils databases used to develop pedotransfer functions are bulk density and organic carbon content. In the following, we show how temporal variations in porosity, pore size distribution and soil water retention can be modelled by tracking the simultaneous effects of various structure‐forming processes on soil pore volumes in a number of user‐defined pore size classes. This idea was first suggested by Gibbs and Reid (1988) as a way to model a dynamic soil macroporosity. However, in the following, we apply this concept to three dynamic pore size classes to make it more generally useful for simulating soil structure dynamics, as different agents and processes (e.g. microbial activity and organic matter dynamics, fauna, roots, tillage etc.) impact different size ranges of pores (see Figure 3). The approach is illustrated using water retention data obtained from two field experiments, one in northern Sweden initiated 63 years ago to study the effects of contrasting crop rotations (Jarvis, Forkman, et al., 2017) and the other in Switzerland, designed to investigate the recovery of soil structure following severe compaction (Keller et al., 2017).

### 4.1 Soil porosity and pore size classes

Our starting point is a fundamental equation for a soil volume *V*_t_ (and corresponding layer thickness Δ*z*) consisting of solid and pore volumes, *V*_s_ and *V*_p_, with the solids comprising organic and mineral matter (*V*_s(o)_ and *V*_s(m)_ respectively) and the pore space partitioned into a static (constant) textural pore volume *V*_p(t)_ and a dynamic structural pore volume *V*_p(s)_:

(1) $$\Delta z=\frac{V_{t}}{A_{\text{xs}}},$$

(2) $$V_{t}=V_{s}+V_{p}=V_{s\left(o\right)}+V_{s\left(m\right)}+V_{p\left(t\right)}+V_{p\left(s\right)},$$

where *A*_xs_ is a nominal cross‐sectional area (e.g. 1 cm^2^). The volume of organic matter can change as the stored mass of SOM changes due to organic amendments, root exudation, biomass growth/decay and mineralization. The volume of structural pores may also vary in response to physical (e.g. swell‐shrink) and biological processes (e.g. root growth, faunal bioturbation and soil aggregation resulting from microbial activity), which then results in changes in the total soil porosity, the pore size distribution and soil water retention. In contrast, the textural pore volume *V*_p(t)_ and the mineral volume *V*_s(m)_ in Equation (2) are both constant and are obtained from user‐defined minimum values of matrix porosity *ϕ*_min_ and soil layer thickness *Δz*_min_ (corresponding to a minimum soil volume) found in a purely mineral soil without any biological activity and organic material (i.e. both *V*_s(o)_ and *V*_p(s)_ are zero):

(3) $$V_{p\left(t\right)}=\phi_{\text{min}}\Delta z_{\text{min}}A_{\text{xs}},$$

(4) $$V_{s\left(m\right)}=V_{p\left(t\right)}\left(\frac{1}{\phi_{\text{min}}}‐1\right).$$

The minimum porosity *ϕ*_min_ can be relatively easily measured for artificial porous materials (see Liu et al., 2019; Shen, Liu, Xu, & Wang, 2019 and references therein). This would probably not be so straightforward for natural soils, although some methods have been proposed (e.g. Fies & Stengel, 1981). In principle, *ϕ*_min_ should vary with particle size distribution, although theoretical particle packing models (e.g. Liu et al., 2019; Shen et al., 2019) suggest that these variations in *ϕ*_min_ should be relatively small. Nimmo (2013) suggested that the closest particle packing in natural soils should result in porosities lying between c. 0.30 and 0.35 cm^3^/cm^3^.

In our approach, the pore volume *V*_p_ is also partitioned into three size classes (hereafter termed macropores, mesopores and micropores) at two fixed pore diameters with the micropore and mesopore volumes (*V*_mic_ and *V*_mes_) together comprising a volume of matrix pores *V*_mat_ and the remaining soil pore volume being composed of macropores (*V*_mac_):

(5) $$V_{p}=V_{\text{mac}}+V_{\text{mat}}=V_{\text{mac}}+V_{\text{mes}}+V_{\text{mic}}.$$

Recognizing the multiscale nature of soil structure (see Figure 3), structural pore space is found in all three size classes, whereas the textural pore space comprises only matrix pores and is partitioned ‘a priori’ into micropore and mesopore fractions (*V*_p(t,mic)_ and *V*_p(t,mes)_):

(6) $$V_{p\left(t,\text{mic}\right)}=f_{t\left(\text{mic}\right)}V_{p\left(t\right)},$$

(7) $$V_{p\left(t,\text{mes}\right)}=\left(1‐f_{t\left(\text{mic}\right)}\right)V_{p\left(t\right)},$$

where *f*_t(mic)_ is the fraction of textural pores in the micropore class, which can be estimated from the soil particle size distribution (e.g. Arya & Heitman, 2015; Arya et al., 1999; Chan & Govindaraju, 2004). Time‐varying porosities can be calculated from the partial volumes as:

(8) $$\phi_{\text{mic}}=\left(\frac{V_{p\left(s,\text{mic}\right)}+V_{p\left(t,\text{mic}\right)}}{V_{t}}\right),$$

(9) $$\phi_{\text{mes}}=\left(\frac{V_{p\left(s,\text{mes}\right)}+V_{p\left(t,\text{mes}\right)}}{V_{t}}\right),$$

(10) $$\phi_{\text{mat}}=\phi_{\text{mic}}+\phi_{\text{mes}},$$

(11) $$\phi_{\text{mac}}=\frac{V_{\text{mac}}}{V_{t}},$$

(12) $$\phi_{t}=\frac{V_{p\left(t\right)}}{V_{t}},$$

(13) $$\phi_{s}=\frac{\left(V_{p\left(s,\text{mic}\right)}+V_{p\left(s,\text{mes}\right)}+V_{\text{mac}}\right)}{V_{t}},$$

(14) $$\phi =\phi_{\text{mat}}+\phi_{\text{mac}}=\phi_{t}+\phi_{s},$$

where *V*_p(s,mic)_ and *V*_p(s,mes)_ are the micropore and mesopore structural pore volumes, *ϕ* is the total porosity, *ϕ*_t_ and *ϕ*_s_ are the textural and structural porosities and *ϕ*_mat_, *ϕ*_mac_, *ϕ*_mic_ and *ϕ*_mes_ are the matrix porosity, macroporosity, microporosity and mesoporosity.

### 4.2 Dynamic soil water retention functions

The model concept described above is directly compatible with the capacity‐type hydrological models employed in some commonly used soil–crop models. This is because these models are based on pore classes defined by porosity, field capacity and wilting point and do not require complete knowledge of the shape of the water retention function. In contrast, dynamic pore volumes for each size class must be translated into a continuous soil water retention function in order to couple the proposed approach to hydrological models based on Richards’ equation. Most widely used water retention functions are unimodal, with their shape described by two parameters, both of which, in principle, may vary with time as the porosity changes (e.g. Assouline, 2006; Stange & Horn, 2005). These functions can account for two dynamic pore classes (e.g. micropores and mesopores) in the soil matrix, but they are not flexible enough to capture the effects of macropores on soil water retention. However, such unimodal functions can easily be extended to account for an additional dynamic pore volume representing soil macroporosity (e.g. Durner, 1994; Fatichi et al., 2020; Jarvis & Larsbo, 2012; Reynolds, 2017).

Unimodal water retention functions can be linked to a dynamic model of matrix pore space comprising two pore size classes by assuming that one of the shape parameters remains constant. We illustrate this taking the widely used empirical model of van Genuchten (1980) as an example. If the residual water content is negligible, water content *θ* (m^3^/m^3^) is given by:

(15) $$\theta =\phi_{\text{mat}}{\left(1+{\left|\alpha \psi \right|}^{n}\right)}^{\frac{1}{n}‐1},$$

where *ψ* (cm) is the soil water pressure head and *α* (cm^−1^) and *n* (−) are shape parameters that reflect the pore size distribution. In a dynamic model for soil water retention, it seems reasonable to suppose that *n* in Equation (15) could be held constant, as it is known to be strongly determined by soil texture (e.g. Vereecken et al., 2010; Wösten, Pachepsky, & Rawls, 2001), while *α* can be allowed to vary, since it should be more influenced by structural porosity (Assouline & Or, 2013). With this assumption, *α* in the van Genuchten (1980) equation can be calculated from:

(16) $$\alpha =\frac{{\left[{\left(\frac{\phi_{\text{mic}}}{\phi_{\text{mat}}}\right)}^{‐\frac{n}{n‐1}}‐1\right]}^{1/n}}{\left|\psi_{\text{mic}/\text{mes}}\right|},$$

where *ψ*_mic/mes_ is the pressure head (cm) defining the size of the largest micropore (i.e. the pressure head at which all mesopores would be air‐filled).

We illustrate the model approach described by Equations ((1), (2), (3), (4), (5), (6), (7), (8), (9), (10), (11), (12), (13), (14), (15), (16)) using data obtained from a long‐term field experiment established in 1956 on a silt loam soil at Offer in northern Sweden (Bolinder, Kätterer, Andrén, & Parent, 2012; Jarvis, Forkman, et al., 2017). The trial includes four treatments that differ with respect to the number of years of grass‐clover ley in a 6‐year crop rotation. Here we discuss data for the two extreme treatments, one with 5 years of grass/clover ley in the rotation (*A*), and the other dominated by arable crops (*D*), with only 1 year of ley. After more than 50 years, the topsoil organic carbon content is c. 50% larger in treatment *A* than *D* (c. 0.032 and 0.022 kg/kg respectively). This is partly because carbon inputs to the soil have been c. 25% larger due to a combination of manure amendment and greater root production, but also because the more frequent tillage in treatment *D* increased organic carbon decomposition rates by c. 10% (Bolinder et al., 2012). Jarvis, Forkman et al. (2017) reported that anecic earthworm species are absent at the site, while the total biomass of endogeic and epigeic earthworms is c. 5 times larger in treatment *A* (1.6 g/m^2^) than in treatment *D* (0.3 g/m^2^).

**FIGURE 4:**
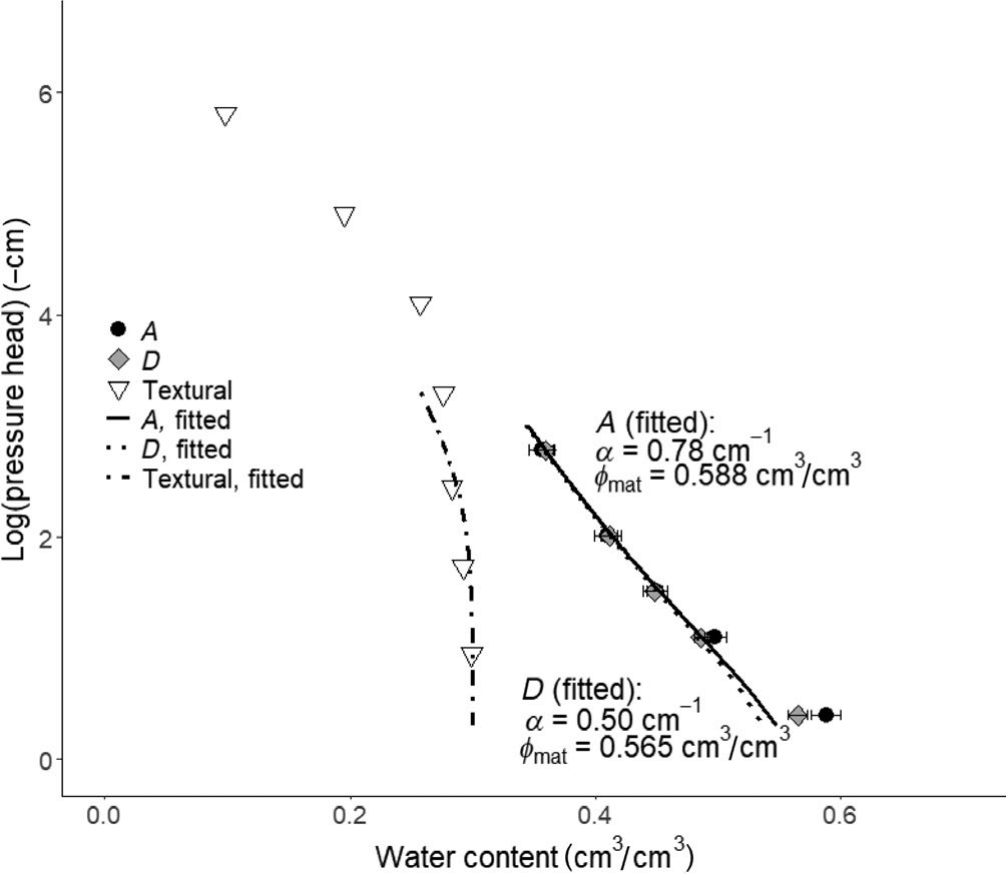
Model concepts illustrated by soil water retention curves measured in two contrasting crop rotations in a long‐term field trial at Offer in northern Sweden. The van Genuchten model (Equations 15 and 16) was fitted to the data with a common *n* value of 1.08, excluding the measurements made at a pressure head of −2.5 cm (i.e. free drainage from saturation). The water retention curve for the textural porosity was predicted by the model of Arya and Heitman (2015) from the measured particle size distribution at the site, assuming a minimum porosity *ϕ*_min_ of 0.3 cm^3^/cm^3^. The maximum micropore diameter is set at 30 μm (i.e. *ψ*_mic/mes_ = −100 cm). Error bars shown on the figures are standard errors of the mean measured water contents

Figure 4 shows soil water retention curves for 12 replicate samples per treatment taken in early November 2019 from the uppermost 10 cm of soil at Offer, alongside one estimated for the textural pore space from measurements of soil particle size distribution using the model described by Arya and Heitman (2015), assuming a minimum porosity *ϕ*_min_ of 0.3 m^3^/m^3^ (Nimmo, 2013). Table 1 shows the pore classes derived from the model fits to the data, with the maximum pore diameter of micropores set to 30 μm. The differences in measured water contents between the treatments are not significant (at *p* = .05) at any pressure head. However, the results suggest that the structural porosity in treatment *A* is slightly larger than in treatment *D*, with pore space >100 µm in diameter being responsible for most of this difference. Capillary bundle theory predicts that the saturated hydraulic conductivity *K*_sat_ should be proportional to the square of the value of *α* in van Genuchten's equation (Mishra & Parker, 1990). This would suggest that *K*_sat_ may be c. 2–3 times larger in treatment *A* than *D*. The actual difference may be larger, since Equation (15) cannot capture the effects of large macropores, which appear to be more abundant in the soil from treatment *A* (Figure 4). Ericson and Mattsson (2000) reported that topsoil *K*_sat_ measured in 1987 was on average c. 10 times larger in treatment *A* than *D*, although this difference was not statistically significant due to large within‐treatment variation. At first sight, the lack of statistically significant differences in soil water retention between the treatments may seem surprising, considering the large differences in organic matter inputs, soil OM content and faunal populations. It may be the case that the effects of enhanced biological activity in treatment *A* at Offer have been partly counteracted by compaction, since the soil is only loosened by tillage 1 year in six, but it is still trafficked several times a year in order to harvest the grass/clover forage crop. A model that can dynamically couple soil physical and biological processes to pore space properties would help to interpret this kind of experimental data, thereby leading to a clearer understanding of the effects of soil and crop management on soil physical degradation and recovery.

**TABLE 1:** Pore classes (m^3^/m^3^) derived from the fits of the van Genuchten (1980) equation to the water retention curves shown in Figure 4, assuming a maximum diameter of micropores of 30 µm (i.e. *ψ*_mic/mes_ = −100 cm; note that macroporosity is assumed zero in both treatments)

Treatment	Textural			Structural			Total		
	*ϕ*_t(mic)_	*ϕ*_t(mes)_	*ϕ*_t_	*ϕ*_s(mic)_	*ϕ*_s(mes)_	*ϕ*_s_	*ϕ*_mic_	*ϕ*_mes_	*ϕ*_mat_
A	0.295	0.005	0.3	0.120	0.168	0.288	0.415	0.173	0.588
D				0.118	0.147	0.265	0.413	0.152	0.565

### 4.3 Linking soil processes to structural pore space dynamics

A simple empirical approach is adopted here to couple the activity of biological agents and processes to the dynamics of the structural pore volumes in the three size classes. We assume that the change in the structural pore volume *V*_p(s,_ *_i_*_)_, in size class *i* is a linear function of the change in the volume of one or more solid constituents in soil, *V*_s(_ *_j_*_)_:

(17) $$\frac{dV_{p\left(s,i\right)}}{dt}=\sum_{j}f_{\mathrm{ij}}\left(\frac{dV_{s\left(j\right)}}{dt}\right),$$

where *t* is time, *V*_s(_ *_j_*_)_ is the volume of a given solid constituent in soil and *f_ij_* are ‘pore‐change’ factors (m^3^ pores/m^3^ solids) which reflect the extent to which a change in *V*_s(_ *_j_*_)_ affects the partial pore volumes in soil. These changes in the volume of solid soil constituents can be caused by, for example, the ingestion and egestion of soil by earthworms and changes in SOM stocks or plant root growth/decay. Temporal variation in the total soil volume is then given by:

(18) $$\frac{dV_{t}}{dt}=\frac{dV_{s}}{dt}+\frac{dV_{p\left(s\right)}}{dt}=\sum_{i}\sum_{j}f_{\mathrm{ij}}\left(\frac{dV_{s\left(j\right)}}{dt}\right)+\sum_{j}\frac{dV_{s\left(j\right)}}{dt}.$$

Table 2 summarizes how the simple concept embodied in Equations (17) and (18) can serve as a framework for empirical modelling of the variations of pore and total soil volumes as a function of the activity of biological agents of structure formation and degradation, with the value of *f_ij_* (with *f_ij_* ≥ −1) depending on the process under consideration. It is easy to show that if the sum of all pore change factors equals −1, then the total soil volume will remain unchanged.

**TABLE 2:** Empirical modelling of dynamic pore and total soil volumes: A simple unified framework to account for the effects of structure‐forming biological agents

Agents of structure formation	Pore ‘change factor’	Comments
**Roots**		Pore compression or blockage
Growth	−1 ≤ *f* ≤ 0	*f* = −1 complete compression or blockage by root growth into existing pores; no change in soil surface elevation
		*f* = 0; no compression or blockage by root growth into existing pores; surface elevation increases
Decay	*f* = −1	Creation of biopores
**Soil fauna**	−1 ≤ *f* ≤ 0	Bioturbation
		Soil ingestion: *f* = −1 without structural change; *f* = 0 with complete collapse (loss of pore volume)
		Soil egestion: *f* = −1 without surface casting; *f* = 0 with no casting in the soil (100% surface casting)
**Microorganisms**	*f* >> 0	Aggregation resulting from microbial decomposition of OM
		Typically, 2 < *f* < 4 (Federer et al., 1993)

With some changes in terminology, the approach described by Equation (17) and illustrated for various biological agents in Table 2, should also be applicable to some of the physical processes driving structure dynamics. For example, in the case of swell/shrink, the matrix pore volume changes in response to changes in the soil water volume, *V*_w_, whereas the volume of solids is constant, so that equation 17 can be rewritten as:

(19) $$\frac{dV_{\text{mat}}}{dt}=f\left(\frac{dV_{w}}{dt}\right),$$

where *f* (0 ≤ *f* ≤ 1) is the slope of the shrinkage characteristic, which depends on soil properties and soil wetness (e.g. Leong & Wijawa, 2015; McGarry & Malafant, 1987; Olsen & Haugen, 1998; Peng & Horn, 2005). Changes in the total soil volume (i.e. layer thickness) and structural (crack) porosity can then be calculated from the shrinkage characteristic and a pore geometry factor that characterizes the dimensionality of shrinkage (Bronswijk, 1988; Kim et al., 1992; Te Brake, van der Ploeg, & de Rooij, 2013). This modelling approach has been successfully applied to predict soil subsidence and cracking under field conditions (e.g. Bronswijk, 1988, 1991; Stewart, Rupp, Abou Najm, & Selker, 2016) and has also been incorporated into tipping bucket type hydrological models as well as those based on Richards’ equation (e.g. Arnold, Potter, King, & Allen, 2005; Bronswijk, 1988).

## 5 CASE STUDY

In the following, we make use of the modelling framework described above to illustrate the likely timescales of recovery from severe traffic compaction resulting from both plant root turnover and soil bioturbation by earthworms. For this particular case, the rate of change of the volume of solids, *V*_s_, in Equation (17) can be written as:

(20) $$\frac{dV_{s}}{dt}=V_{t}\left\{\left(\frac{R_{g}‐R_{d}}{\gamma_{r}}\right)+\left(\frac{E_{c}‐E_{i}}{\gamma_{s}}\right)\right\},$$

where *R*_g_ and *R*_d_ are the rates of root biomass growth and decay respectively (g cm^−3^ year^−1^), *γ*_r_ is the density of roots (g/cm^3^), *E*_c_ and *E*_i_ are the rate of casting within the soil and the earthworm ingestion rate respectively (g soil cm^−3^ year^−1^) and *γ*_s_ is the soil specific density (g/cm^3^).

We first show the results of long‐term simulations in which we assume that earthworms do not cast at the soil surface and that the casting rate and root biomass are both at steady‐state (i.e. *E*_c_ = *E*_i_ and *R*_g_ = *R*_d_). With these assumptions, *V*_s_, *V*_p(s)_, *V*_t_ and thus the total soil porosity all remain constant. However, root turnover and earthworm bioturbation may affect the pore size distribution, even if the porosity is unchanged. Combining Equations (17) and (20) gives:

(21) $$\frac{d\phi_{s\left(i\right)}}{dt}=\left[\left(f_{g\left(i\right)}‐f_{d\left(i\right)}\right)\left(\frac{B_{r}\tau_{r}}{\gamma_{r}}\right)\right]+\left[\left(f_{c\left(i\right)}‐f_{s\left(i\right)}\right)\left(\frac{\gamma_{b}\tau_{s}}{\gamma_{s}}\right)\right],$$

where *ϕ*_s(_ *_i_*_)_ is the structural porosity in class *i*, *B*_r_ is the root biomass (g/cm^3^), *τ*_r_ is the root turnover rate (year^−1^), *γ*_b_ is the bulk density (g/cm^3^) and *τ_s_* is the turnover rate of the soil mass by earthworms (year^−1^) and the subscripts g, d, c and s on the pore‐change factors *f* refer to root growth, root decay, earthworm casting and soil ingestion by earthworms respectively. Values for these 12 individual pore‐filling factors (see Table 3) were derived by assuming that:

**TABLE 3:** Pore‐change factors in Equation 21

Pore class	Pore‐change factors			
	Root production		Earthworm bioturbation	
	Growth, *f*_g_	Decay, *f*_d_	Casting, *f*_c_	Ingestion, *f*_s_
Macropores	0	−*f*_r(c)_	$‐\left(1+\varepsilon_{\text{casts}}\right)$	$‐\left(1+\varepsilon \right)$
Mesopores	$‐\left(\frac{\phi_{\text{mes}}}{\phi_{\text{mes}}+\phi_{\text{mic}}}\right)$	*f*_r(c)_ – 1	$\left(1‐f_{\text{casts}\left(\text{mic}\right)}\right)\varepsilon_{\text{casts}}$	$\left(\frac{\phi_{\text{mes}}}{\phi_{\text{mes}}+\phi_{\text{mic}}}\right)\varepsilon$
Micropores	$‐\left(\frac{\phi_{\text{mic}}}{\phi_{\text{mes}}+\phi_{\text{mic}}}\right)$	0	$f_{\text{casts}\left(\text{mic}\right)}\varepsilon_{\text{casts}}$	$\left(\frac{\phi_{\text{mic}}}{\phi_{\text{mes}}+\phi_{\text{mic}}}\right)\varepsilon$
Sum	−1	−1	−1	−1

- Ingestion of a volume of soil matrix by earthworms creates an equivalent macropore volume, while the loss of structural mesopores and micropores by ingestion is proportional to their relative volumes.
- Egestion of earthworm casts within the soil fills in existing macropores and creates new mesopores and micropores.
- Root growth compresses structural micropores and mesopores proportionally to their relative volumes, but has no effect on macropores.
- Root growth into macropores (Table 2) is neglected.
- Root decay creates new macropores and structural mesopores, depending on the fraction of the root biomass comprising coarse and fine roots.

Substituting the values for *f* listed in Table 3 into Equation (21) gives:

(22a) $$\frac{d\phi_{\text{mac}}}{dt}=\left[f_{r\left(c\right)}\left(\frac{B_{r}\tau_{r}}{\gamma_{r}}\right)\right]+\left[\left(\varepsilon ‐\varepsilon_{\text{casts}}\right)\left(\frac{\gamma_{b}\tau_{s}}{\gamma_{s}}\right)\right],$$

(22b) $$\begin{array}{cc} \frac{d\phi_{s\left(\text{mes}\right)}}{dt} & =\left[\left(\left(1‐f_{r\left(c\right)}\right)‐\left(\frac{\phi_{s\left(\text{mes}\right)}}{\phi_{s\left(\text{mes}\right)}+\phi_{s\left(\text{mic}\right)}}\right)\right)\left(\frac{B_{r}\tau_{r}}{\gamma_{r}}\right)\right]\\ & \;+\left[\left(\left(1‐f_{\text{casts}\left(\text{mic}\right)}\right)\varepsilon_{\text{casts}}‐\left(\frac{\phi_{s\left(\text{mes}\right)}}{\phi_{s\left(\text{mes}\right)}+\phi_{s\left(\text{mic}\right)}}\right)\varepsilon \right)\left(\frac{\gamma_{b}\tau_{s}}{\gamma_{s}}\right)\right], \end{array}$$

(22c) $$\begin{array}{cc} \frac{d\phi_{s\left(\text{mic}\right)}}{dt} & =\left[‐\left(\frac{\phi_{s\left(\text{mic}\right)}}{\phi_{s\left(\text{mes}\right)}+\phi_{s\left(\text{mic}\right)}}\right)\left(\frac{B_{r}\tau_{r}}{\gamma_{r}}\right)\right]\\ & \;+\left[\left(\left(f_{\text{casts}\left(\text{mic}\right)}\varepsilon_{\text{casts}}\right)‐\left(\frac{\phi_{s\left(\text{mic}\right)}}{\phi_{s\left(\text{mes}\right)}+\phi_{s\left(\text{mic}\right)}}\right)\varepsilon \right)\left(\frac{\gamma_{b}\tau_{s}}{\gamma_{s}}\right)\right], \end{array}$$

where *ϕ*_s(mes)_ and *ϕ*_s(mic)_ are the structural mesoporosity and microporosity respectively, *ε* is the void ratio of the soil matrix ($=\phi_{\text{mat}}/1‐\phi$), *ε*_casts_ is the void ratio of the earthworm casts, *f*_casts(mic)_ is the fraction of the pore space in the casts comprising micropores and *f*_r(c)_ is the proportion of coarse roots. This model is sufficiently simple that steady‐state solutions can be obtained:

(23) $$\phi_{\text{mat}}=\left(1‐\phi \right)\left[\varepsilon_{\text{casts}}‐\left(\frac{B_{r}f_{\left(r(c\right)}}{\gamma_{r}}\right)\left(\frac{\tau_{r}}{\tau_{s}}\right)\left(\frac{\gamma_{s}}{\gamma_{b}}\right)\right],$$

(24) $$\phi_{s\left(\text{mic}\right)}=\frac{\varepsilon_{\text{casts}}f_{\text{casts}\left(\text{mic}\right)}}{\left(\frac{1}{\phi_{\text{mat}}‐\phi_{\min}}\right)\left\{\left(\frac{B_{r}}{\gamma_{r}}\right)\left(\frac{\tau_{r}}{\tau_{s}}\right)\left(\frac{\gamma_{s}}{\gamma_{b}}\right)+\left(\frac{\phi_{\text{mat}}}{1‐\phi }\right)\right\}},$$

(25) $$\phi_{\text{mic}}=\phi_{s\left(\text{mic}\right)}+f_{t\left(\text{mic}\right)}\phi_{\min},$$

(26) $$\phi_{\text{mes}}=\phi_{\text{mat}}‐\phi_{\text{mic}},$$

(27) $$\phi_{\text{mac}}=\phi ‐\phi_{\text{mat}}.$$

Equation (23) shows that there is a theoretical possibility of this model producing a negative soil matrix porosity at steady‐state, which is physically impossible. However, putting typical parameter values into Equation (23) suggests that this is highly unlikely to occur in practice. Equation (23) also suggests that in the absence of roots (i.e. on bare soil plots with *B*_r_ = 0), the matrix porosity at steady‐state will equal the porosity of the earthworm casts, which has also been inferred from field experiments (Blanchart, 1992; Blanchart et al., 1993, 1997).

**FIGURE 5:**
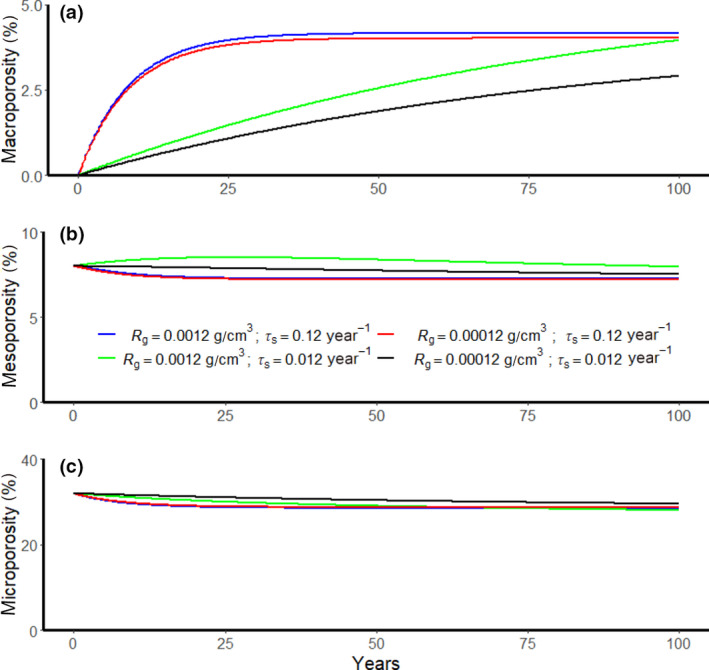
(a‐c) The evolution of soil porosity simulated by the model described by Equation ((22a), (22b), (22c)) for four combinations of root turnover and earthworm bioturbation rates (see also Table 3)

Figure 5a–c shows the results of 100 year simulations with the transient model described by Equation ((22a), (22b), (22c)) and the parameter values shown in Table 4, assuming initial conditions of zero macroporosity and 0.32 and 0.08 cm^3^/cm^3^ for *ϕ*_mic_ and *ϕ*_mes_ respectively. Four scenario simulations are shown consisting of combinations of high and low bioturbation and root production rates. Figure 5a–c shows that soil macroporosity reaches an equilibrium within c. 20–30 years at high earthworm bioturbation rates, whereas recovery from compaction as a result of root production is predicted to be much slower, and is still not complete after a century. Note that for these simulations, we have not attempted to translate variations in pore volumes into a dynamic water retention function, since large changes only occur in the macropore region.

**TABLE 4:** Parameter values used in scenario simulations of the recovery of soil structure following severe compaction as a result of root production and earthworm activity

Parameter	Value
Porosity, *ϕ*, cm^3^/cm^3^	0.4
Minimum porosity, *ϕ*_min_, cm^3^/cm^3^	0.3
Micropore fraction of textural porosity, *f*_t(mic)_	0.8
Particle density, *γ*_s_, g/cm^3^	2.7
Root density, *γ*_r_, g/cm^3^	1.2
Fraction coarse roots, *f*_c(r)_	0.2
Root production, *R*_g_ (=*B_r_ τ*_r_), g cm^−3^ year^−1^	0.0012; 0.00012^a^
Bioturbation rate, *τ*_s_, year^−1^	0.12; 0.012
Fraction of micropores in casts, *f*_casts(mic)_	0.8
Cast void ratio, *ε*_casts_	0.6

^a^ Equivalent to 30% of an above‐ground biomass production of 10 and 1 t ha^−1^ year^−1^ for an annual crop added to a soil layer 25 cm in thickness.

**FIGURE 6:**
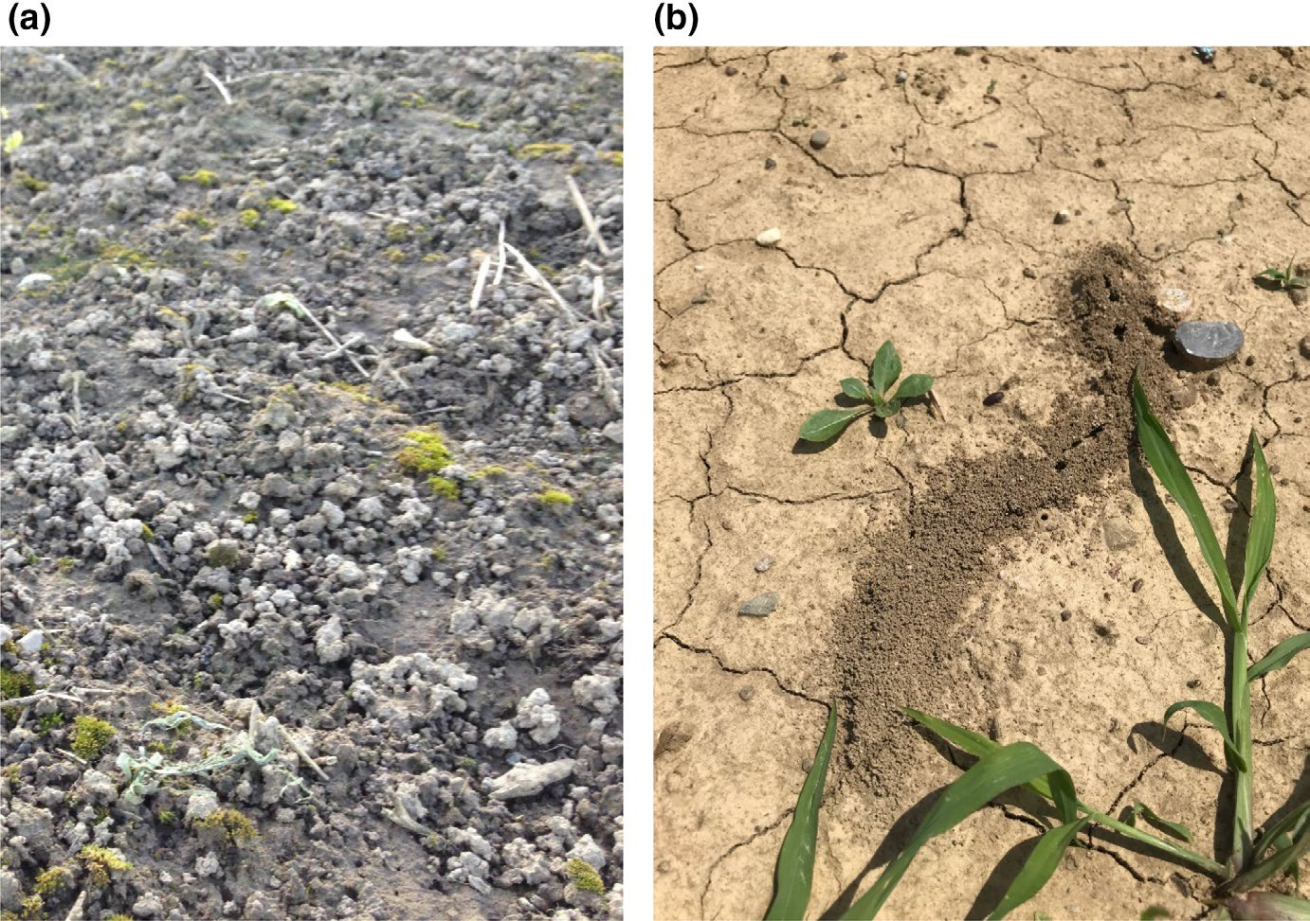
Photographs of surface casting by earthworms (a) and ants (b) on the bare soil plots at the compaction recovery experiment at Agroscope, Zürich, Switzerland (Keller et al., 2017)

We now show the results of a preliminary test of this model using measurements made on samples taken at 0–30 cm depth from bare soil plots monitored during a 4 year period following severe compaction by heavy field traffic in a field experiment at Zürich in Switzerland (Keller et al., 2017). As the soil was free from plants, except for a few weeds, we ignored the effects of roots and only modelled faunal bioturbation. The model was calibrated against data on bulk density, porosity and water contents measured at pressure heads of −30 and −100 cm. Thus, micro‐, meso‐ and macroporosity were assumed to comprise pores smaller than 30, 30–100 μm and larger than 100 μm in equivalent diameter respectively. For unknown reasons, data from the control treatment also showed some significant variations between sampling occasions. Thus, to reveal long‐term trends related to compaction recovery, the measured data on the compacted plots was multiplied by the ratio of the initial value to the current value on the control plots. It was also apparent from the data that the topsoil porosity had increased following the initial compaction. Seasonal variations in porosity due to swelling and shrinking might be expected at this site, because the topsoil has a clay content of 25%–28% (Keller et al., 2017). However, this should not result in any systematic change in porosity during a 4 year period. Instead, field observations suggest that this observed trend can be attributed to the deposition of soil at the surface, primarily as a result of earthworm casting, but also to some extent by burrowing ants (Figure 6). We therefore modified the pore‐change factor for earthworm casting such that only a fraction is egested into macropores and the remaining fraction *f*_surf_ is cast at the soil surface. In this case, the total soil volume *V*_t_ will increase if *f*_surf_ > 0, as the sum of *f*_c_ (Table 3) for the three pore regions is then larger than −1. It should be noted here that we still assume uniform properties in the bioturbated soil layer. The turnover rate due to bioturbation *τ*_s_ in Equations ((21), (22a), (22b), (22c)) and ((21), (22a), (22b), (22c)) can be expressed as:

(28) $$\tau_{s}=\frac{I_{r}E_{\text{bio}}}{\gamma_{b}},$$

where *I*_r_ is the soil ingestion rate (g soil g^−1^ biomass year^−1^) and *E*_bio_ is the earthworm biomass (g/cm^3^). Combining Equation ((22a), (22b), (22c)) (with *B*_r_ = 0) and Equation (28) and accounting for surface casting gives the changes in the structural pore volumes due to bioturbation as:

(29) $$\frac{dV_{\text{mac}}}{dt}=V_{t}\left(\frac{I_{r}E_{\text{bio}}}{\gamma_{s}}\right)\left\{\varepsilon ‐\varepsilon_{\text{casts}}+f_{\text{surf}}\left(1+\varepsilon_{\text{casts}}\right)\right\},$$

(30) $$\frac{dV_{p\left(s,\text{mes}\right)}}{dt}=V_{t}\left(\frac{I_{r}E_{\text{bio}}}{\gamma_{s}}\right)\left[\left\{\left(1‐f_{\text{casts}\left(\text{mic}\right)}\right)\varepsilon_{\text{casts}}\right\}‐\left\{\left(\frac{V_{p\left(s,\text{mes}\right)}}{V_{p\left(s,\text{mes}\right)}+V_{p\left(s,\text{mic}\right)}}\right)\varepsilon \right\}\right],$$

(31) $$\frac{dV_{p\left(s,\text{mic}\right)}}{dt}=V_{t}\left(\frac{I_{r}E_{\text{bio}}}{\gamma_{s}}\right)\left[\left\{f_{\text{casts}\left(\text{mic}\right)}\varepsilon_{\text{casts}}\right\}‐\left\{\left(\frac{V_{p\left(s,\text{mic}\right)}}{V_{p\left(s,\text{mes}\right)}+V_{p\left(s,\text{mic}\right)}}\right)\varepsilon \right\}\right].$$

The time‐course of total porosity and micro‐, meso‐ and macroporosities is calculated with Equations ((1), (2), (3), (4), (5), (6), (7), (8), (9), (10), (11), (12), (13), (14), (29), (30), (31)) and ((1), (2), (3), (4), (5), (6), (7), (8), (9), (10), (11), (12), (13), (14), (29), (30), (31)), while the time‐varying bulk density is given by:

(32) $$\gamma_{b}=\gamma_{s}\left(1‐\phi \right).$$

Satisfactory results with this model could only be obtained by assuming larger surface casting rates in more compact soil, something that has also been found in previous studies (e.g. Buck, Langmaack, & Schrader, 2000; Joschko et al., 1989; Jouquet et al., 2012; Kretzschmar, 1991; Zund et al., 1997). A simple one‐parameter threshold function for *f*_surf_ was adopted such that casting into macropores decreases as the macroporosity decreases below a threshold value *ϕ*_mac(c)_:

(33) $$f_{\text{surf}}=\text{max}\left[0,\left\{1‐\left(\frac{\phi_{\text{mac}}}{\phi_{\text{mac}\left(c\right)}}\right)\right\}\right].$$

With this model formulation, it is easy to show that *ϕ*_mac(c)_ and *ε*_casts_ define the steady‐state (equilibrium) soil macroporosity and matrix porosity respectively, while *f*_casts(mic)_, *ϕ*_min_ and *f*_t(mic)_ control the partitioning of the steady‐state matrix porosity between micropores and mesopores.

**FIGURE 7:**
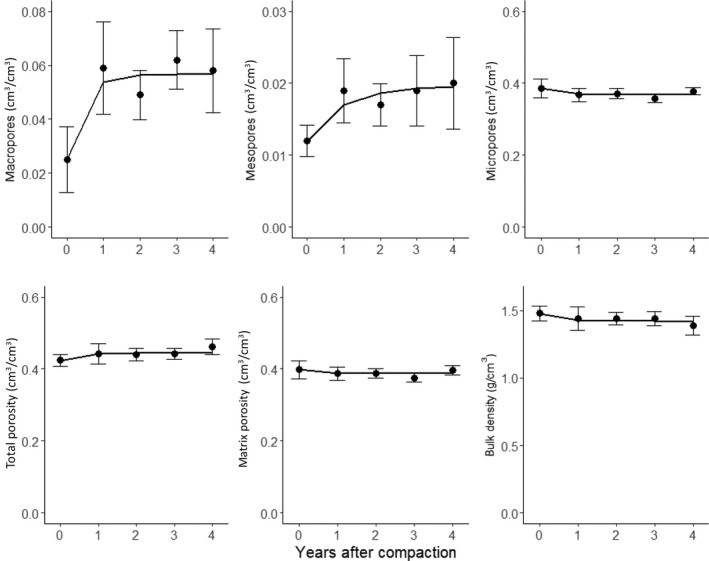
Comparison of model simulations (lines, given by Equations (1), (2), (3), (4), (5), (6), (7), (8), (9), (10), (11), (12), (13), (14), (29), (30), (31), (32), (33) and (1), (2), (3), (4), (5), (6), (7), (8), (9), (10), (11), (12), (13), (14), (29), (30), (31), (32), (33) with *I*_r_ = 2.79 g soil g^−1^ biomass day^−1^, *ϕ*_mac(t)_ = 0.057, *ε*_casts_ = 0.714 and *f*_casts(mic)_ = 0.845) with observed soil physical properties on bare soil plots at the compaction recovery experiment (Keller et al., 2017; bars are standard deviations)

Initial values of the state variables were set according to measurements made immediately after compaction. We used the average total earthworm biomass (i.e. including endogeic, epigeic and anecic earthworm species) measured on three sampling occasions after compaction (Keller et al., 2017; T. Keller, unpublished data) to estimate *E*_bio_ (=655 kg/ha at 0–30 cm depth, equivalent to 218 g/m^3^). Measurements of initial bulk density and porosity were used to estimate the particle density *γ*_s_ (=2.56 g/cm^3^). The minimum porosity *ϕ*_min_ was fixed at 0.35 cm^3^/cm^3^ (Nimmo, 2013), while the micropore fraction of textural pores *f*_t(mic)_ was fixed at 0.966 by assuming that the mesoporosity that was measured immediately following compaction comprised only textural pores. The four other parameters in the model (*I*_r_, *ε*_casts_, *f*_casts(mic)_ and *ϕ*_mac(c)_) were estimated by calibration using the Powell conjugate gradient method (Powell, 2009). The analysis was repeated 100 times with different starting values for the parameters to check the uniqueness of the optimized values. Figure 7 shows that the calibrated model satisfactorily reproduced the temporal changes in micro‐, meso‐ and macroporosity and bulk density observed at the field site. The calibrated value of the soil ingestion rate (=2.79 g soil g^−1^ biomass day^−1^) is at the high end of the range of values reported in field experiments for temperate geophagous species (e.g. Curry & Schmidt, 2007). The model simulations suggest that topsoil macro‐ and mesoporosity had largely recovered from compaction within 3 years as a result of earthworm bioturbation.

## 6 CONCLUDING REMARKS

More than 25 years ago, Cresswell et al. (1992) concluded that … “*simulation models incorporating well established physical laws are effective tools in the study of soil structural effects on the field water regime. Their application, however, is constrained by insufficient knowledge of the fundamental hydraulic properties of … soils and how they are changing in response to our land management*.” This is still the case today (Vereecken et al., 2016; Vogel et al., 2018). The simple concept and methodology outlined in this paper shows promise as one way to integrate the effects of the individual agents of structure dynamics within a single unified modelling framework in order to assess the typical timescales of soil degradation and recovery. Some important feedback effects in the soil–plant system, whereby changes in soil structure also impact the biological agents of soil structure formation (e.g. plant root growth, soil fauna populations; see Figure 1) have not been discussed here. The dynamic two‐way nature of these interactions should be explicitly addressed in future modelling efforts (e.g. Dignac et al., 2017; Smithwick, Lucash, McCormack, & Sivandran, 2014; Vereecken et al., 2016; Vogel et al., 2018). Furthermore, in this paper, we have mostly neglected the effects of physical processes and focused on mechanisms and models for soil structure dynamics generated by the biological processes that are important for good soil physical quality. Future model developments should consider both the physical and biological processes driving soil structure dynamics, including their significant interactions.

Laboratory experiments under controlled conditions have helped to shed light on the fundamental mechanisms driving structure changes in soil by enabling the study of the effects of individual processes (i.e. root growth or earthworm activity) in isolation. This is especially the case for experiments that utilize modern imaging techniques to quantify changes in soil structure (Hallett et al., 2013; Vereecken et al., 2016). The new modelling concepts presented here may also help to focus attention on those components of the soil system where data are still lacking and where further experimental research is therefore needed. Long‐term field experiments designed to investigate the effects of alternative management practices on crop production and the environment are also a valuable resource. However, with only a few exceptions (e.g. Keller et al., 2017), soil physical and hydraulic properties have not been monitored in such long‐term field trials, presumably because they are implicitly considered to be constant. Judicious and simultaneous exploitation of both of these experimental approaches should help to support the development and parameterization of new soil–crop models that can account for the dynamics of soil structure and its effects on key processes in the soil–plant system. Ultimately, this may lead to more reliable predictions of the impacts of soil degradation on soil properties and ecosystem services, thereby helping to support the development of cost‐efficient strategies for sustainable crop production and the restoration of degraded soils, in line with the UN 2030 agenda for sustainable development.

## Supporting information

Table S1

## Data Availability

The data that support the findings of this study are available in the supplementary material of this article.
