# Supplementary material for: A framework for modelling soil structure dynamics induced by biological activity
Source: Glob Chang Biol. 2020 Aug 23;26(10):5382–403. doi: 10.1111/gcb.15289 (PMC7539949; doi:10.1111/gcb.15289)
Supplement: Supplementary file 1 — Table S1 [file GCB-26-5382-s001.docx]

**Supplementary information**

Table S1. Raw data (means and standard deviations) from the compaction recovery experiment at Agroscope, Zurich, Switzerland used in the modelling case study, the results of which are shown in Figure 7. Detailed information on the study site and the data can be found in Keller et al. (2017).

| Months after compaction | ^1^Treatment | Bulk density  (g cm^-3^) | Porosity  (cm^3^ cm^-3^) | Macroporosity (cm^3^ cm^-3^) | Mesoporosity (cm^3^ cm^-3^) |
| --- | --- | --- | --- | --- | --- |
| 0 | FC | 1.476 (0.022) | 0.424 (0.009) | 0.025 (0.004) | 0.012 (0.002) |
| 12 | FC | 1.416 (0.042) | 0.455 (0.016) | 0.068 (0.010) | 0.016 (0.002) |
| 24 | FC | 1.508 (0.038) | 0.419 (0.014) | 0.041 (0.009) | 0.016 (0.003) |
| 36 | FC | 1.445 (0.040) | 0.445 (0.014) | 0.039 (0.008) | 0.014 (0.004) |
| 48 | FC | 1.395 (0.045) | 0.470 (0.017) | 0.042 (0.019) | 0.021 (0.003) |
|  |  |  |  |  |  |
| 0 | NC | 1.337 (0.055) | 0.482 (0.024) | 0.079 (0.022) | 0.023 (0.006) |
| 12 | NC | 1.313 (0.031) | 0.494 (0.011) | 0.093 (0.018) | 0.020 (0.002) |
| 24 | NC | 1.403 (0.041) | 0.460 (0.017) | 0.068 (0.023) | 0.022 (0.002) |
| 36 | NC | 1.346 (0.038) | 0.484 (0.017) | 0.050 (0.021) | 0.018 (0.007) |
| 48 | NC | 1.339 (0.027) | 0.491 (0.011) | 0.057 (0.016) | 0.025 (0.002) |

^1^ FC = full compaction, NC = no compaction

Keller, T., Colombi, T., Ruiz, S., Pogs Manalili, M., Rek, J., Stadelmann, V., … Or, D. (2017). Long-term soil structure observatory for monitoring post-compaction evolution of soil structure. *Vadose Zone Journal*, doi:10.2136/vzj2016.11.0118
